# Transport of Twelve Coumarins from Angelicae Pubescentis Radix across a MDCK-pHaMDR Cell Monolayer—An *in Vitro* Model for Blood-Brain Barrier Permeability

**DOI:** 10.3390/molecules200711719

**Published:** 2015-06-25

**Authors:** Yan-Fang Yang, Wei Xu, Wei Song, Min Ye, Xiu-Wei Yang

**Affiliations:** State Key Laboratory of Natural and Biomimetic Drugs, Department of Natural Medicines, School of Pharmaceutical Sciences, Peking University Health Science Center, Peking University, No. 38, Xueyuan Road, Haidian District, Beijing 100191, China; E-Mails: yangyanfang@bjmu.edu.cn (Y.-F.Y.); high-xu@163.com (W.X.); sw-yy1990@163.com (W.S.); yemin@bjmu.edu.cn (M.Y.)

**Keywords:** Angelicae Pubescentis Radix, coumarins, MDCK-pHaMDR cells, blood brain barrier, permeability, structure-permeability relationship

## Abstract

Angelicae Pubescentis Radix (APR), a widely used traditional Chinese medicine, is reported to have central nervous system activities. The purpose of this study was to characterize the blood-brain barrier permeability of twelve coumarins from APR including umbelliferone (**1**), osthol (**2**), scopoletin (**3**), peucedanol (**4**), ulopterol (**5**), angepubebisin (**6**), psoralen (**7**), xanthotoxin (**8**), bergapten (**9**), isoimperatorin (**10**), columbianadin (**11**), and columbianetin acetate (**12**) with an *in vitro* model using a MDCK-pHaMDR cell monolayer. The cell monolayer was validated to be suitable for the permeation experiments. The samples’ transports were analyzed by high performance liquid chromatography and their apparent permeability coefficients (*P_app_*) were calculated. According to the *P_app_* value, most coumarins could be characterized as well-absorbed compounds except for **4**, **10** and **11** which were moderately absorbed ones, in concentration-dependent and time-dependent manners. The results of P-glycoprotein (P-gp) inhibitor (verapamil) experiments showed that the transport of coumarin **4** was affected by the transport protein P-gp. Sigmoid functions between permeability log(*P*_*app* AP-BL_*MW^0.5^) and log D (at pH 7.4) were established to analyze the structure-activity relationship of coumarins. The results provide useful information for discovering the substance basis for the central nervous system activities of APR, and predicting the permeability of other coumarins through BBB.

## 1. Introduction

Phytochemicals have been used as promising therapeutic agents for encephalopathy in recent years. Angelicae Pubescentis Radix (APR, the roots of *Angelica pubescens* Maxim. f. *biserrata* Shan et Yuan), known as Duhuo in Chinese, is one of the most widely used traditional Chinese medicines. Pharmacological studies indicate that APR possesses anti-inflammatory [1], analgesic [2], anticancer [3] and platelet aggregation inhibitory [4] activities. Other studies show that APR also has central nervous system (CNS) activities [5] of inhibiting the apoptosis of brain cells [6], protecting from H_2_O_2_-induced SH-SY5Y cells injury [7], and being effective in treating Alzheimer’s disease (AD) [8]. Coumarins are the main active components of APR, and more than 60 coumarins have isolated and identified from APR [9]. Previous studies have reported that many of these coumarins have biological CNS activities. For instance, scopoletin has anti cholinergic- and age-impaired memory ameliorative activities [10,11]; osthole possesses the ability to protect cortical neurons and SH-SY5Y cells against β-amyloid peptide (Aβ) injury [12] and traumatic brain injury [13]; isoimperatorin [14], psoralen [15] and xanthotoxin [16,17] can inhibit both acetylcholinesterase activity for the treatment of AD and brain monoamine oxidase activity for the treatment of affective disorders. The above studies suggest that coumarins are the prime ingredients contributing to the CNS activity of APR. The intestinal absorptions of main coumarins from APR, such as umbelliferone, osthol, columbianadin, columbianetin acetate, psoralen, bergapten, xanthotoxin and isoimperatorin, have been studied with human colon adenocarcinoma cell line (Caco-2) cell monolayer in our group, and all of them are defined as well or moderately intestinal absorbed compounds [18,19,20]. Therefore, it is necessary to study the penetration abilities of the main coumarins of APR through the blood-brain barrier (BBB) for accessing its CNS pharmacological activities.

BBB permeability is one of the key determinants for CNS exposure and the kinetics evaluation of drugs [21]. BBB is a strong barrier between the blood and brain parenchyma, providing the stable microenvironment that is critical for complex neural function and protecting the CNS from chemical insult and damage [22]. Due to the high complexity of both passive penetration and active transport processes, cell cultures are the favored tools for BBB drug penetration prediction. Among them, the multidrug resistant Madin-Darby Canine kidney (MDCK-MDR1) cell line has been proved to be a simple, quick and proper surrogate BBB model on the basis of the electron microscopical morphology, transepithelial electrical resistance (TEER), transport functionality and P-glycoprotein (P-gp) function [23,24]. Under the cell culture conditions, MDCK-MDR1 cells form a monolayer after eight days and exhibit high TEER because of the tight junction expression [23]. MDCK-MDR1 cell monolayer has been used to study the BBB permeability of different active constituents from natural medicines, such as ginkgolides [25], decursin and decursinol angelate [26]. 

**Figure 1:**
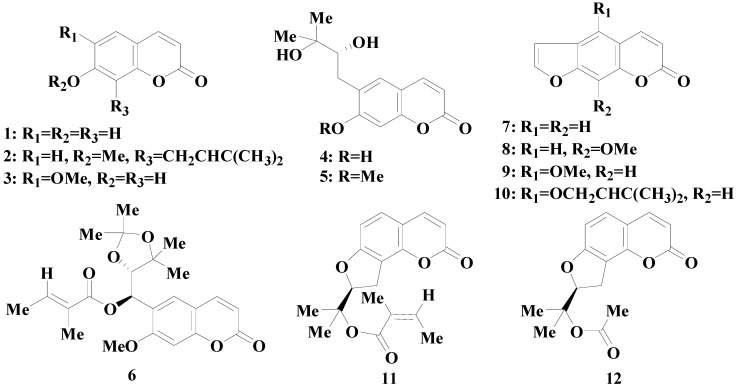
Chemical structures of coumarins from Angelicae Pubescentis Radix: umbelliferone (**1**), osthol (**2**), scopoletin (**3**), peucedanol (**4**), ulopterol (**5**), angepubebisin (**6**), psoralen (**7**), xanthotoxin (**8**), bergapten (**9**), isoimperatorin (**10**), columbianadin (**11**) and columbianetin acetate (**12**).

In this paper, MDCK-pHaMDR cell line, derived from the parental MDCK cell line after infection with the MDR1 virus produced by the cell line PA-12-MDR1/A1 [27], was obtained from National Institutes of Health (NIH, Bethesda, MD, USA), and utilized to study the BBB penetration of twelve coumarins from APR (chemical structures shown in Figure 1), along with the time- and concentration-dependent absorption. The samples were analyzed by high performance liquid chromatography (HPLC) and the transport parameter apparent permeability coefficient (*P_app_*) was calculated. The aim is to discover the substance basis for the CNS activities of APR and the possible transport mechanisms of coumarins through BBB, together with the BBB permeability prediction of coumarins.

## 2. Results and Discussion

### 2.1. Validation of the MDCK-pHaMDR Cell System

The applicability of MDCK-pHaMDR cell monolayer as *in vitro* BBB model was validated. MTT assays showed that all coumarins at the maximum test concentration of 125 µM exerted no significant influence on cell viability.

As shown in Table 1, the TEERs of test cell monolayer were all above 1000 Ω·cm^2^ in accord with the reported values [26]. Caffeine and atenolol (as the well- and poor-transported marker by passive diffusion) were run from the apical side (AP) to basolateral side (BL) across the MDCK-pHaMDR cell monolayer, and *P_app_* values were calculated as 4.36 × 10^−5^ cm/s and 7.61 × 10^−7^ cm/s, respectively. The results were comparable to reported values [26] and the applicability of the cell monolayer as *in vitro* BBB model was verified.

**Table 1:** The bidirectional *P_app_* values of twelve coumarins in the MDCK-pHaMDR cell monolayer.

Compound	*P*_*app* AP-BL_ (×10^−6^ cm/s)	*P*_*app* BL-AP_ (×10^−6^ cm/s)	*P*_*app* BL-AP_/*P*_*app* AP-BL_	LogD (pH 7.4)	TEER (Ω·cm^2^)
**1**	35.86 ± 2.28	32.79 ± 1.25	0.91	1.44	1321 ± 129
**2**	23.79 ± 1.44	20.37 ± 3.04	0.86	4.08	1548 ± 104
**3**	37.10 ± 1.97	27.38 ± 3.51	0.96	1.65	1586 ± 194
**4**	9.91 ± 0.21	18.97 ± 0.38	1.91	0.63	1595 ± 93
**5**	22.36 ± 4.02	26.66 ± 2.94	1.19	1.09	1664 ± 190
**6**	20.85 ± 1.32	18.73 ± 1.02	0.90	3.40	1347 ± 110
**7**	27.34 ± 2.35	26.75 ± 2.75	0.98	2.08	1549 ± 82
**8**	26.21 ± 3.54	23.34 ± 1.67	0.89	2.17	1298 ± 161
**9**	43.62 ± 2.13	37.38 ± 5.81	0.86	2.17	1595 ± 173
**10**	7.29 ± 0.17	3.73 ± 0.33	0.51	3.7	1615 ± 136
**11**	10.42 ± 0.95	7.12 ± 0.12	0.68	4.28	1429 ± 152
**12**	38.34 ± 1.72	33.74 ± 6.35	0.88	2.54	1463 ± 119
caffeine (10 µM)	43.58 ± 4.86				1635 ± 53
Atenolol (200 µM)	0.76 ± 0.16				1573 ± 91

*P*_*app* AP-BL_: transport from AP to BL side; *P*_*app* BL-AP_: transport from BL to AP side; *P*_*app* BL-AP_/*P*_*app* AP-BL_: efflux ratio of *P*_*app* BL-AP_ to *P*_*app* AP-BL_. The concentration of all coumarins was 50 µM. The incubation time was up to 90 min. Data are means ± S.D. (*n* = 6).

Rhodamine 123 (Rh123), a typical probe substrate for P-gp, has been widely used in P-gp researches. When co-incubated with the P-gp inhibitor verapamil, Rh123 efflux was inhibited with the efflux ratio of *P*_*app* BL-AP_/*P*_*app* AP-BL_ decreasing significantly from 4.47 to 1.58, and the intracellular accumulation of Rh123 was 4.45-fold higher than that of verapamil-absent group (Table 2). All results indicated that P-gp was stably expressed in the MDCK-pHaMDR cell and played obvious efflux effect on drug transport.

**Table 2:** Rh123 transportation and intracellular accumulation in MDCK-pHaMDR cell.

Compound	*P_app_* (×10^−7^ cm/s)		*P*_*app* BL-AP_/*P*_*app* AP-BL_	Intracellular Accumulation Fluorescence Intensity
	AP-BL	BL-AP		
Rh123	11.81 ± 0.56	52.74 ± 2.09	4.47 ± 0.24	34.51 ± 1.18
Rh123 + 100 µM verapamil	5.36 ± 0.41	8.51 ± 1.43	1.58 ± 0.208 *	153.64 ± 3.28 *

*P*_*app* AP-BL_: transport from AP to BL side; *P*_*app* BL-AP_: transport from BL to AP side; *P*_*app* BL-AP_/*P*_*app* AP-BL_: efflux ratio of *P*_*app* BL-AP_ to *P*_*app* AP-BL_. The concentration of Rh123 was 10 µM. Data are means ± S.D. (*n* = 4). * *p* < 0.01 *vs.* verapamil-absent group.

### 2.2. Bidirectional Transport Determination

The permeation samples were examined by HPLC since coumarins had good ultraviolet absorptions and the maximum wavelength was chosen for each compound. Rapid, simple and reliable HPLC methods had been established for the analysis of twelve coumarins. The standard calibration curves were constructed by plotting peak area (y) *vs.* concentration (x, µM). Regression equation, coefficient correlation (r) of the methods for twelve coumarins were shown as follow: y = 14.82x + 4.21 (0.9998, for **1**), y = 10.47x −021.85 (0.9992, for **2**), y = 12.26x − 10.59 (0.9997, for **3**), y = 11.95x + 8.62 (0.9993, for **4**), y = 18.44x + 3.58 (0.9998, for **5**), y = 16.32x − 14.86 (0.9998, for **6**), y = 23.50x − 28.19 (0.9995, for **7**), y = 12.59x + 0.44 (0.9997, for **8**), y = 10.76x + 9.68 (0.9995, for **9**), y = 7.46x + 3.70 (0.9998, for **10**), y = 12.58x − 22.87 (0.9993, for **11**), y = 18.25x − 6.53 (0.9997, for **12**), respectively. The concentration range of the calibration curve was 0.5–150 µM for **4**, **5**, **7**, **8**, 1–150 µM for **1**, **2**, **3**, **6**, **9**, **10**, **12**, and 2–150 µM for **11**, respectively. The intraday and interday precision of the methods for twelve test coumarins were less than 3.41% and 3.79%, respectively, and the accuracies of the methods were between 85.18% and 107.71%. The concentrations after three freeze-thaw cycles changed from 95.71% to 103.97%, or less than ±5% range. The data, summarized in Supplemental Materials Table S1, proves the HPLC methods were feasible.

In general, drugs with high *P_app_* (>1 × 10^−5^ cm/s) can be well-absorbed, while those with low *P_app_* (<1 × 10^−6^ cm/s) are poorly absorbed [23]. The bidirectional *P_app_* values for coumarins **1**–**12** have been summarized in Table 1. The *P*_*app* AP-BL_ values of **1**–**3**, **5**–**9** and **11**–**12** were at the level of 10^−5^ cm/s, similar to that of caffeine, so they were classified as well-absorbed compounds through the BBB. Coumarins **4** and **10** were assigned to the moderately absorbed compounds, since their *P*_*app* AP-BL_ values of 10^–6^ cm/s laid between the levels of caffeine and atenolol. The possible pathway of most test coumarins was passive transport because the ratios of *P*_*app* BL-AP_/*P*_*app* AP-BL_ were less than 2.0 [23]. For compounds **4**, **10** and **11**, the ratios of *P*_*app* BL-AP_/*P*_*app* AP-BL_ were high near 2.0 or low near 0.5, so P-gp inhibitor verapamil was further used to verify their absorption pathway.

To check the intracellular accumulation and mass balance, the recoveries of twelve coumarins (total amounts of the compounds in both sides of the insert and intracellular) were measured. The test coumarins had high recoveries of >85% and low intracellular accumulations of <7%, except compound **11** (Supplemental Materials Table S2). As for compound **11**, the relative low recovery (64%–68%) and high intracellular accumulation (9%–22%) were clearly different from those of the other coumarins.

### 2.3. Time- and Concentration-Dependent Permeation

**Figure 2:**
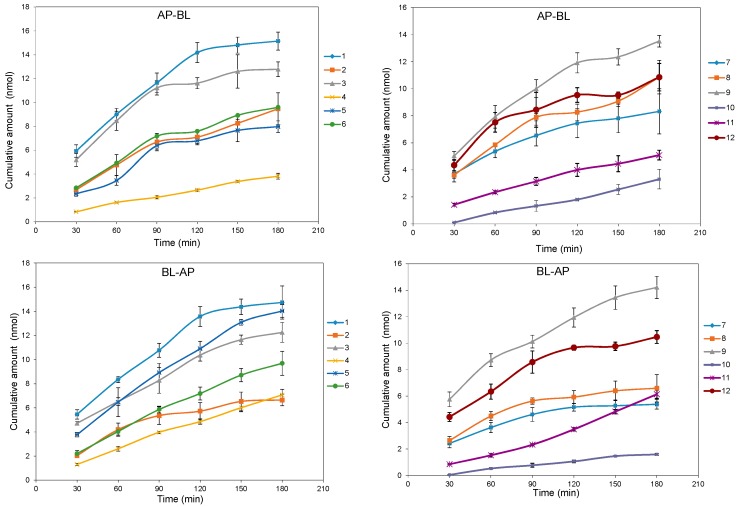
The bidirectional transport cumulative amounts of twelve coumarins in MDCK-pHaMDR cell monolayer as a function of time at 50 μM. Data are the mean ± S.D. (*n* = 6).

**Figure 3:**
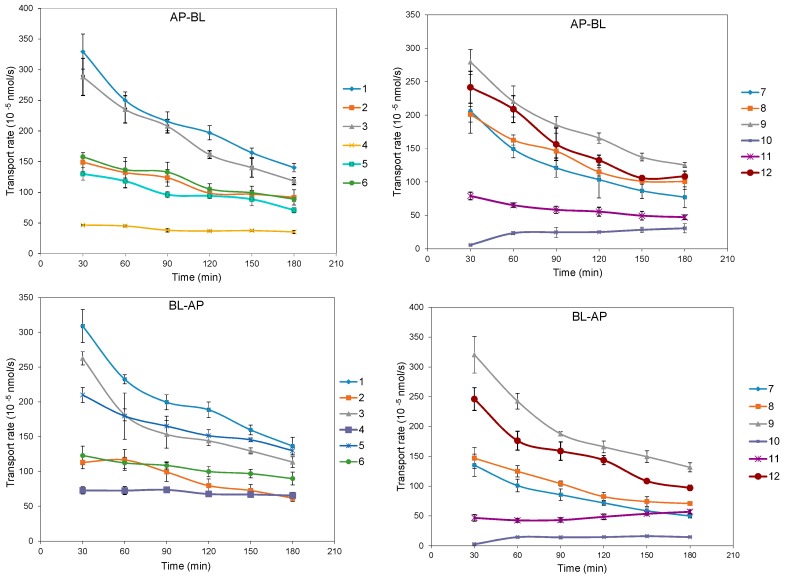
The bidirectional transport rate of twelve coumarins in MDCK-pHaMDR cell monolayer as a function of time at 50 μM. Data are the mean ± S.D. (*n* = 6).

**Figure 4:**
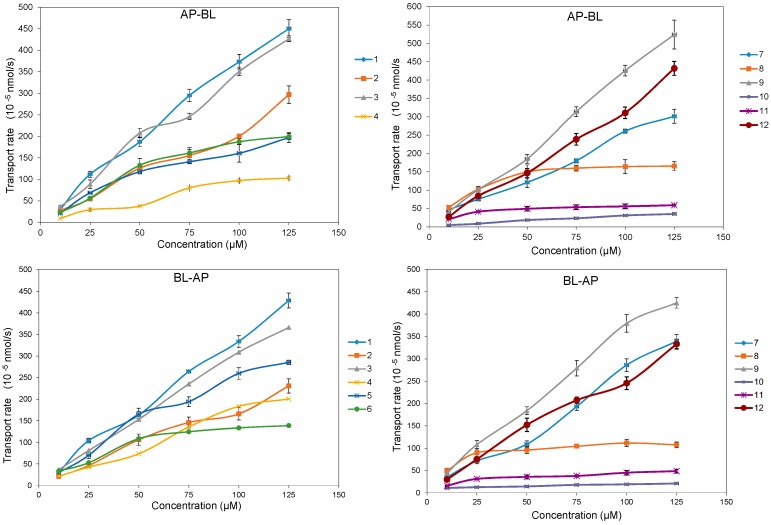
The bidirectional transport rate of twelve coumarins in MDCK-pHaMDR cell monolayer as a function of concentration at 10–125 μM. Data are the mean ± S.D. (*n* = 6).

The bidirectional time-permeation curves of twelve test coumarins at 50 µM are presented in Figure 2 and Figure 3. Concentration difference between membrane sides is one of the important features of passive diffusion. When the concentration difference of test compounds between the receiver side and donor side reaches a certain degree, the transport rate will slow down. Both AP-BL and BL-AP transport cumulative amounts of all compounds at 50 µM increased almost linearly with time, while the transport rates of all compounds except **10** decreased linearly with time, according to the transport balance of concentration saturation. The slight increase transport rate of **10** in 60 min maybe relate with a long time to reach concentration saturation because of its smallest bidirectional *P_app_*. The bidirectional transport rates of most test coumarins increased linearly within the concentration range of 10–125 µM (Figure 4), except that the transport rates of **4** and **8** in AP-BL direction and **6**, **8**, **11** in BL-AP direction had slower increase in high concentrations (from 75 µM). For these compounds, since the linear trends were obvious in the concentrations under 75 µM but close to slow platform from 75 to 125 µM, a possible explanation was that the drug saturation on the receiver side slowed down the transport at higher concentrations.

### 2.4. Transport Inhibition by Verapamil

**Figure 5:**
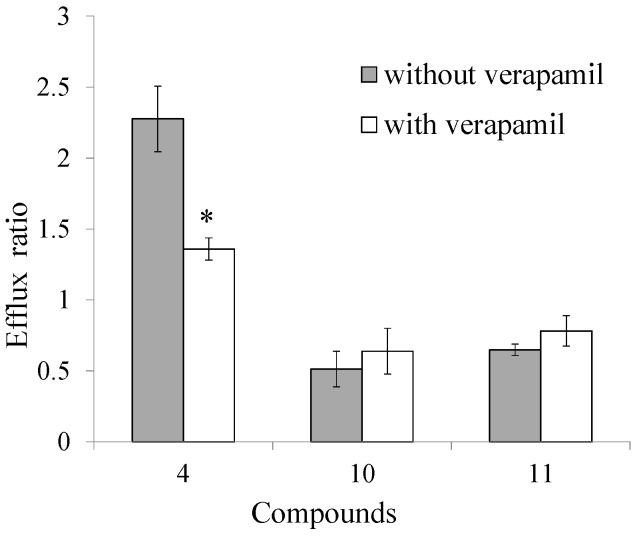
The efflux ratios of coumarins **4**, **10**, **11** in MDCK-pHaMDR cell in the absence or presence of 100 μM verapamil. * *p* < 0.01 *vs.* verapamil absent group.

Since the ratios of *P*_*app* BL-AP_/*P*_*app* AP-BL_ of compounds **4**, **10** and **11** were high near 2.0 or low near 0.5, verapamil at 100 µM was used as a selective P-gp inhibitor during the transport experiment. When bidirectional transports of selected coumarins toghter with 100 µM verapamil were studied, the ratio of *P*_*app* BL-AP_/*P*_*app* AP-BL_ of compound **4** significantly decreased by 40.35%, while there were no significant changes of compounds **10** and **11** (Figure 5). The results revealed that P-gp related efflux mechanism was involved in the transport of compound **4**, while P-gp had no effect on the absorption process of coumarins **10** and **11**.

### 2.5. Structure-Permeability Relationship Analysis

To analyze the structure-permeability relationship, *P_app_* values of the test coumarins were compared. There were significant differences in *P_app_* values between **1** and **2**, as well as **7** and **10** when contrasted, which indicated that the straight chain substitutions on the coumarin nucleus may decrease the coumarin penetration through the BBB. The *P_app_* value similarities between **1** and **3**, as well as **7** and **8**, showed that the methoxyl group substitutent on the aromatic ring had no effect on the coumarin penetration through the BBB, but the different substituent positions of the methoxyl group may have obvious effects on the coumarin penetrations according to the *P_app_* comparison between **8** and **9**. The *P_app_* difference between **4** and **5** revealed that methoxylation of a hydroxyl group can obviously promote the penetration of coumarins. The comparison between **11** and **12** indicated that an angeloyl substituent maybe give lower *P_app_* values than an acetyl group. In addition, the *P_app_* difference between **1** and **7** showed that a furan ring substituted at C_6_ and C_7_ of the aromatic ring may decrease the coumarin absorption across the BBB.

**Figure 6:**
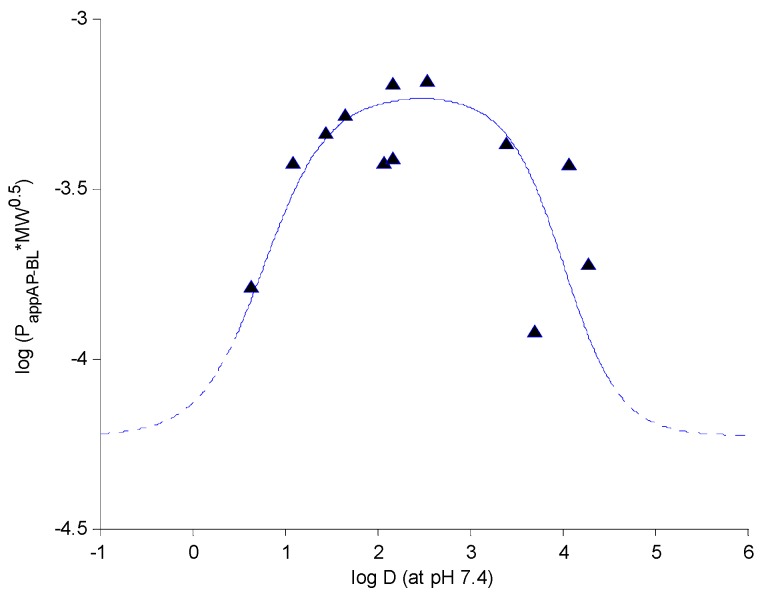
The relationship between permeability log (*P*_*app* AP-BL_*MW^0.5^) and log D (at pH 7.4) of twelve coumarins.

Physicochemical characters, such as the logarithm of distribution-coefficient (log D), the logarithm of partition-coefficient (log P), and polar surface area, are generally utilized for the prediction of drug permeability. In this study, it was notable that the coumarin penetrations across MDCK-pHaMDR cell monolayer were not well correlated with their log P. Theoretical passive drug absorption [28] and experimental transport across intestinal epithelial cells [29] have proved that lipophilicity has a close relationship with the tansepithelial permeability of compounds. Our previous works have demonstrated the sigmoid relationship between permeability log (*P*_*app* AP-BL_*MW^0.5^) and log D of neolignans [30] and coumarins [20] in Caco-2 cell monolayer. In this study, log D (at pH 7.4) was chosen as the index of lipophilicity, calculated with Pallas 3.3.2.6 ADME/Tox Software (CompuDrug, Bal Harbor, FL,USA) (Table 1). Two sigmoid functions linked at log D 2.5 were resulted by plotting log (*P*_*app* AP-BL_*MW^0.5^) *vs.* log D (at pH 7.4) with Matlab (R2013a, MathWorks, Natick, MA, USA) (Figure 6) and the equations are listed as follow:

(1) $$f\left(\text{x}\right)=\{\begin{array}{c} \frac{1}{1+{\text{e}}^{-2.9\text{x}-2.25}}\;-\;4.227\;\text{x}\leq 2.5\\ \frac{1}{1+{\text{e}}^{3.3\text{x}-13.25}}\;-\;4.227\;\text{x}>2.5 \end{array}$$

The sigmoid curve suggested that the BBB transepithelial permeabilities of coumarins (compounds **1**, **3**, **5**, **6**, **7**, **8**, **9**, **12**) with moderate log D (1.0–3.5) were relatively high and less affected by the change of log D, while the penetrations of those with higher log D (3.5–4.5) (**2**, **10**, **11**) or lower log D (<1.0) (**4**) decreased rapidly when log D changed. The result indicated that too high or too low lipophilicity may result in low BBB permeability, which agreed with the researches of intestinal permeability [29]. Therefore log D as the index of lipophilicity was important and useful for predicting drug BBB permeability.

## 3. Experimental Section

### 3.1. Chemicals and Reagents

Twelve coumarins, umbelliferone (**1**), osthol (**2**), scopoletin (**3**), peucedanol (**4**), ulopterol (**5**), angepubebisin (**6**), psoralen (**7**), xanthotoxin (**8**), bergapten (**9**), isoimperatorin (**10**), columbianadin (**11**), columbianetin acetate (**12**), were isolated from APR and characterized in our group [31,32]. The purities were determined to be above 98.0%. Colchicine, caffeine, atenolol, dimethylsulfoxide (DMSO) were purchased from Sigma-Aldrich (St. Louis, MO, USA). Methanol (MeOH) was of HPLC grade (J. T. Backer, Center Valley, PA, USA). Milli-Q water (Millipore, Bedford, MA, USA) was used throughout the study. Other chemicals were of analytical grade.

### 3.2. Cell Culture

MDCK-pHaMDR cell line was a gift from Dr. Michael M. Gottesman (NIH, Bethesda, MD, USA). Dulbecco’s Mocified Eagle’s Medium (DMEM), Fetal bovine serum (FBS), trypsin, penicillin, streptomycin and other culture media and supplements were obtained from Gibco (Life Science Technologies, Carlsbad, CA, USA). Reagents for Hank’s Balanced Salts Solution (HBSS) were from Beijing Chemical Works (Beijing, China). 3-(4,5-Dimethyl-2-thiazolyl)-2,5-diphenyl-2*H*-tetrazolium bromide (MTT), Rh123 and verapamil were purchased from Sigma-Aldrich (St. Louis, MO, USA). 12 Wells Transwell^®^ plates with polycarbonate inserts (3 µm pore size and 12 mm in diameter) and 6 Wells plates were obtained from Corning Costar (Cambridge, MA, USA). The cytotoxicity of the test compounds on the MDCK-pHaMDR cells was determined with the MTT assay on a Thermo Multiskan MK3 Automated Microplate Reader (Thermo-Labsystems, Franklin, MA, USA).

The cells were cultured at 37 °C in 5% CO_2_, using DMEM with 4.5 g of glucose per liter and 1 mM sodium pyruvate supplemented with 10% FBS, 5 mM l-glutamine, 50 units/mL penicillin and 50 µg/mL streptomycin. Cells were harvested with 0.25% trypsin-EDTA and seeded onto the Transwell insert filter at a density of about 8 × 10^4^ cells/mL. After growing for 8 days, the cells reached confluence and full differentiation for the transport experiments [26]. All cells used in this study were between passages 8 and 30.

### 3.3. Transport Experiments

10 mM stock solutions of test compounds were prepared in DMSO and diluted with HBSS to the desired concentrations before transport experiments. HBSS (pH 7.4) was used as a transport medium. After washing the MDCK-pHaMDR cell monolayer twice with prewarmed HBSS medium, HBSS medium was preserved to prepare samples of the calibration curves. Then the experiments were carried out by replacing the transport medium in the AP side (0.5 mL, for absorption transport) or BL side (1.5 mL, for efflux transport) with test compounds (50 µM). The plates were shaked at 55 rpm for 90 min at 37 °C in a water bath. Samples were collected from both sides of the cell monolayer, then immediately frozen, lyophilized and preserved below −20 °C. To measure the intracellular accumulation amounts of different coumarins, the cell monolayers were extracted after transport assays with 200 µL 70% MeOH (*v*/*v*) for 20 min, then centrifuged and preserved below −20 °C.

### 3.4. Standard Conditions of MDCK-pHaMDR Cell Monolayer

The integrity and transport capacity of the MDCK-pHaMDR cell monolayer were examined by measuring the TEER with an epithelial voltohmmeter (EVOM, World Precision Instrument, Sarasota, FL, USA). Only cell monolayer with a TEER above 1000 Ω·cm^2^ was used for the transport assay [26]. Standard compounds, caffeine and atenolol were run as the active and passive transport marker, respectively.

The P-gp expression level in the MDCK-pHaMDR cell monolayer was examined by Rh123 transport and intracellular accumulation experiments. Rh123 (10 µM) bidirectional transports with or without the presence of verapamil (100 µM) were undertaken as described above, and the lyophilized permeation samples were dissolved in a proper volume of MeOH. The fluorescence at 485 nm (excitation wavelength) and 538 nm (emission wavelength) was determined on a Cary Eclipse Fluorescence spectrophotometer (Varian Inc., Palo Alto, CA, USA). To investigate intracellular accumulations of Rh123 as a probe substrate for P-gp, MDCK-pHaMDR cells were seeded on the 6 Wells plates at a density of about 1.0 × 10^5^ cells/mL and grew for 48 h. Before the uptake experiments, cells were washed with PBS two times and pre-incubated with serum-free DMEM or 100 µM verapamil for 20 min. Rh123 with the final concentration of 10 µM was added, and cells were incubated with or without the presence of verapamil for 60 min at 37 °C. Then cells were washed with ice-cold PBS three times, and lysed with 0.1% (*v*/*v*) Triton X-100 for 15 min at 37 °C [33]. The fluorescence of Rh123 in cell lysates was determined as described above.

### 3.5. Time- and Concentration-Dependent Transport Experiments

The permeable coumarins were chosen to study the time- and concentration-dependence permeability across the MDCK-pHaMDR cell monolayer. To observe the time-dependence of the selected coumarin, test compounds at 50 µM were added to either AP or BL side of the inserts for absorption transport (AP-BL) or efflux transport (BL-AP), and incubated for 30, 60, 90, 120, 150 and 180 min. In the concentration-dependent transport study, 10, 25, 50, 75, 100, and 125 µM of certain coumarins were added to either AP or BL side and incubated for 90min. Transport experiments were undertaken as described above.

### 3.6. Verapamil Inhibition of Transport

The verapamil inhibition experiments were carried out for compounds **4**, **10** and **11**. The cells were pre-incubated with 100 µM verapamil for 30 min before drug transport assays. Then the inhibitor was added to both sides of the membrane and the coumarins (**4**, **10** and **11**) at 50 µM were added to AP or BL side. Coumarin transport experiments were undertaken as described above and the efflux ratios of *P_app_*_BL-AP_/*P_app_*_AP-BL_ were calculated.

### 3.7. HPLC Analysis

An Agilent 1100 series HPLC system (Agilent Technologies, Palo Alto, CA, USA) was used with an analytical Diamonsil^®^ C_18_ column (250 mm × 4.6 mm, 5 µm, Dikma, Beijing, China) equipped with a C_18_ guard column (8 mm × 4 mm, 5 µm, Dikma). The mobile phase was composed of MeOH-H_2_O (*v*/*v*) in 68:32 for **1**, 90:10 for **2**, 53:47 for **3**, 55:45 for **4**, 66:34 for **5**, 88:12 for **6**, 75:25 for **7**, 80:20 for **8**, 70:30 for **9**, 92:8 for **10**, 75:25 for **11**, 78:22 for **12**, and the UV detection was at 320 nm for **1**, 330 nm for **2**, **4**, **5**, **11** and **12**, 344 nm for **3**, 325 nm for **6**, 250 nm for **7**, 310 nm for **8** and **10**, 300 nm for **9**. The flow rate was 1.0 mL/min. Elution peaks were monitored and the peak areas were used to calculate the compound concentrations.

To evaluate linearity, calibration curves were prepared and assayed. Accuracy and precision were assessed by determining quality control (QC) samples at three concentration levels (5, 50 and 150 µM) on three different consecutive days. The recoveries at three QC levels were also determined. The freeze-thaw stabilities were assessed by analyzing the QC samples undergoing three freeze (−20 °C)–thaw (room temperature) cycles. To determine the corresponding coumarins, the lyophilized permeation samples were dissolved in a suitable volume of MeOH, thoroughly vortex-mixed for 1 min and then centrifuged at 16,000× *g* for 20 min. An aliquot of 20 µL supernatant solution was used for HPLC assay. To measure the intracellular accumulation amount of different coumarins, the aliquots of cell extractions were determined too.

### 3.8. Data Analysis

*P_app_* in AP-BL or BL-AP direction of each coumarin was calculated from the following equation:

(2) $$P_{app}=\frac{\text{dQ}}{\text{dt}\cdot \text{A}\cdot {\text{C}}_{0}}\;[\text{cm}/\text{s}]$$

where Q is the accumulation quantity of the compound in the receiver side (μmol), dQ/dt is the linear appearance rate of the compound in the receiver side (μmol/s), C_0_ is the initial concentration in the donor side (μM), and A is the surface area of the membrane insert (cm^2^). Data were expressed as the mean ± SD.

## 4. Conclusions

In conclusion, most test coumarins can be transported across the MDCK-pHaMDR cell monolayer by passive diffusion and are well or moderately absorbed. The compound structure may have an effect on the coumarin penetration capacity, and log D values may have important relationship with the permeability through the BBB. The present results provide some useful information for studying the CNS activity of APR and predicting the penetration capacity of coumarins through the BBB. Based on the results, our next study objective will be to observe the brain distributions of the main coumarins of APR and carry out a comparison between the *in vivo* and *in vitro* absorption characteristics of coumarins. In addition, according to the polarized expression of P-gp in the MDCK-pHaMDR cell line and our previous works on log D related permeability studies, the results could be explained on the basis of a P-gp efflux-related transport mechanism and lipophilicity-permeability relationship analysis. Since there are other enzymes, proteins and receptors to cover the whole functional features of the BBB, there are some limitations of the findings and further experiments about other factors of BBB are expected.

## Supplementary Materials

HPLC method validations, together with the cell accumulation and total recovery of coumarin transport are available as Supplementary Materials, which may be accessed at: http://www.mdpi.com/1420-3049/20/07/11719/s1.
