# Supplementary material for: Transport of Twelve Coumarins from Angelicae Pubescentis Radix across a MDCK-pHaMDR Cell Monolayer—An in Vitro Model for Blood-Brain Barrier Permeability
Source: Molecules. 2015 Jun 25;20(7):11719–32. doi: 10.3390/molecules200711719 (PMC6332004; doi:10.3390/molecules200711719)
Supplement: Supplementary file 1 [file molecules-20-11719-s001.pdf]

Supplemental Data

Table S1. Precision, accuracy, recovery and stability of HPLC method validations for twelve coumarins.

Compound	QC (μM)	Precision RSD (%)		Accuracy (%)		Recovery (%)	Stability (%)
		Intra-Day	Inter-Day	Intra-Day	Inter-Day		
**1**	5	0.70	2.68	96.51 ± 1.34	97.42 ± 3.27	101.97 ± 0.54	100.51 ± 3.24
	50	1.01	1.53	91.73 ± 1.84	91.62 ± 1.55	95.74 ± 1.92	101.34 ± 1.24
	150	1.82	3.02	90.19 ± 4.09	91.73 ± 4.89	88.36 ± 4.00	103.01 ± 1.74
**2**	5	1.74	1.81	88.33 ± 1.80	87.68 ± 1.86	95.89 ± 6.97	96.28 ± 2.34
	50	1.40	2.70	85.95 ± 1.39	87.33 ± 2.32	89.09 ± 2.34	96.64 ± 3.32
	150	3.04	3.79	85.57 ± 2.13	87.35 ± 2.95	86.05 ± 3.29	100.15 ± 4.93
**3**	5	1.67	3.31	101.34 ± 0.68	99.17 ± 3.76	98.65 ± 8.13	98.38 ± 1.43
	50	2.09	2.67	85.18 ± 1.29	86.36 ± 2.89	92.92 ± 4.09	99.62 ± 0.71
	150	1.67	2.96	98.95 ± 2.00	96.41 ± 2.99	89.53 ± 2.86	100.79 ± 0.75
**4**	5	0.96	1.05	104.12 ± 1.09	96.41 ± 2.99	96.56 ± 6.00	100.53 ± 2.64
	50	0.45	0.53	88.35 ± 0.92	86.53 ± 3.38	86.86 ± 3.38	98.94 ± 1.55
	150	0.14	1.83	91.27 ± 0.37	92.24 ± 1.69	88.22 ± 1.51	99.74 ± 1.52
**5**	5	0.62	1.77	97.09 ± 3.12	96.73 ± 3.42	102.87 ± 3.31	97.51 ± 3.70
	50	1.12	1.89	93.13 ± 0.87	94.22 ± 1.22	87.11 ± 6.49	99.09 ± 1.19
	150	3.03	3.23	90.02 ± 2.64	89.92 ± 2.77	92.82 ± 2.72	100.48 ± 0.96
**6**	5	2.02	2.83	107.71 ± 2.24	107.38 ± 2.55	87.68 ± 1.85	100.40 ± 1.36
	50	3.41	3.63	99.75 ± 3.31	100.37 ± 3.90	89.13 ± 3.04	100.03 ± 0.78
	150	3.36	3.50	89.88 ± 2.79	90.09 ± 3.13	85.26 ± 2.96	99.76 ± 1.15
**7**	5	0.67	0.89	95.28 ± 0.56	95.51 ± 1.62	84.03 ± 1.12	98.18 ± 1.30
	50	2.36	3.11	92.15 ± 1.92	93.62 ± 3.93	92.03 ± 2.69	103.97 ± 0.62
	150	2.67	3.29	91.13 ± 1.83	89.88 ± 3.44	85.16 ± 6.24	99.63 ± 3.90
**8**	5	1.05	1.48	98.27 ± 1.05	99.65 ± 2.09	87.22 ± 1.13	98.15 ± 0.54
	50	2.83	2.87	100.20 ± 2.75	101.90 ± 2.91	84.52 ± 1.52	98.17 ± 0.19
	150	2.23	2.38	101.16 ± 2.26	101.51 ± 2.79	84.37 ± 2.08	100.18 ± 1.20

Table S1. *Cont.*

Compound	QC (μM)	Precision RSD (%)		Accuracy (%)		Recovery (%)	Stability (%)
		Intra-Day	Inter-Day	Intra-Day	Inter-Day		
**9**	5	1.24	2.12	90.56 ± 1.03	89.84 ± 2.03	89.46 ± 2.43	103.06 ± 1.07
	50	1.01	1.21	90.17 ± 0.19	90.36 ± 1.67	92.53 ± 3.09	95.71 ± 0.63
	150	1.35	2.06	88.86 ± 0.21	85.80 ± 3.49	88.28 ± 2.12	97.18 ± 2.96
**10**	5	1.80	2.95	102.14 ± 2.19	98.84 ± 3.01	88.51 ± 1.90	104.09 ± 0.54
	50	1.12	1.44	103.11 ± 2.04	104.71 ± 2.39	93.23 ± 2.61	103.31 ± 1.79
	150	0.99	1.85	98.31 ± 0.59	96.69 ± 3.25	86.02 ± 2.06	96.21 ± 2.29
**11**	5	1.82	2.74	104.03 ± 1.90	103.08 ± 2.83	85.75 ± 1.71	101.25 ± 2.29
	50	0.09	3.75	104.59 ± 3.10	105.58 ± 3.96	85.05 ± 2.52	100.11 ± 0.81
	150	1.01	3.52	97.61 ± 0.99	99.08 ± 3.48	86.85 ± 7.84	100.83 ± 2.47
**12**	5	0.76	1.95	89.06 ± 0.60	88.62 ± 0.67	92.96 ± 0.63	101.47 ± 2.58
	50	1.13	2.19	87.65 ± 0.99	86.93 ± 2.18	96.34 ± 1.58	102.19 ± 2.15
	150	0.23	2.96	87.28 ± 2.21	85.51 ± 2.72	85.90 ± 2.10	98.88 ± 2.87

Table S2. Cell accumulation and total recovery of twelve coumarins in the bidirectional transport experiment.

Compound	AP→BL		BL→AP	
	CA (%)	TR (%)	CA (%)	TR (%)
**1**	N.D.	99.34 ± 1.59	N.D.	96.70 ± 3.22
**2**	6.96 ± 1.50	104.93 ± 5.15	3.51 ± 0.68	95.63 ± 1.75
**3**	N.D.	87.47 ± 3.30	N.D.	92.15 ± 3.16
**4**	N.D.	102.79 ± 4.07	N.D.	102.81 ± 2.71
**5**	N.D.	93.57 ± 1.89	N.D.	94.93 ± 1.28
**6**	1.40 ± 0.16	86.17 ± 5.70	0.83 ± 0.09	88.83 ± 2.47
**7**	4.63 ± 0.13	89.89 ± 6.49	1.79 ± 0.03	94.73 ± 3.85
**8**	N.D.	95.17 ± 4.79	N.D.	88.69 ± 3.13
**9**	N.D.	99.15 ± 0.54	N.D.	107.04 ± 1.19
**10**	5.38 ± 1.14	90.19 ± 3.08	1.65 ± 0.36	86.64 ± 2.89
**11**	21.49 ± 4.56	67.63 ± 5.00	9.44 ± 1.01	64.11 ± 3.63
**12**	0.94 ± 0.11	96.97 ± 3.36	0.34 ± 0.03	96.54 ± 1.23

CA (Cell accumulation): the percent of the coumarins that accumulated in the cell monolayer after transport experiment relative to the initial amount. TR (Total recovery): the percent of the total amount of the coumarins recovered from both sides of the inserts and intracellular accumulation in MDCK-pHaMDR cell monolayer relative to the initial amount. N.D.: not detected. The concentration of all coumarins was 50 μM. The incubation time was up to 90 min. Data are means ± S.D. (n = 6).
